# Effectiveness of Two Universal Angiosperm Probe Sets Tested In Silico for Caryophyllids Taxa with Emphasis on Cacti Species

**DOI:** 10.3390/genes13040570

**Published:** 2022-03-24

**Authors:** Delil A. Chincoya, Sofía Solórzano

**Affiliations:** 1 Laboratorio de Ecología Molecular y Evolución, UBIPRO, FES Iztacala, Universidad Nacional Autónoma de México, Avenida de los Barrios 1, Los Reyes Iztacala, Tlalnepantla de Baz, Estado de México 54090, Mexico; 2 Posgrado en Ciencias Biológicas, UNAM, FES Iztacala, Circuito de Posgrados, Ciudad Universitaria, Coyoacán, Ciudad de México 04510, Mexico

**Keywords:** cactaceae, caryophyllids, phylogenomics, target enrichment, universal probe sets

## Abstract

In angiosperms, huge advances in massive DNA sequencing technologies have impacted phylogenetic studies. Probe sets have been developed with the purpose of recovering hundreds of orthologous loci of targeted DNA sequences (TDS) across different plant lineages. We tested in silico the effectiveness of two universal probe sets in the whole available genomes of Caryophyllids, emphasizing phylogenetic issues in cacti species. A total of 870 TDS (517 TDS from Angiosperm v.1 and 353 from Angiosperms353) were individually tested in nine cacti species and *Amaranthus hypochondriacus* (external group) with ≥17 Gbp of available DNA data. The effectiveness was measured by the total number of orthologous loci recovered and their length, the percentage of loci discarded by paralogy, and the proportion of informative sites (PIS) in the alignments. The results showed that, on average, Angiosperms353 was better than Angiosperm v.1 for cacti species, since the former obtained an average of 275.6 loci that represent 123,687 bp, 2.48% of paralogous loci, and 4.32% of PIS in alignments, whereas the latter recovered 148.4 loci (37,683 bp), 10.38% of paralogous loci, and 3.49% of PIS. We recommend the use of predesigned universal probe sets for Caryophyllids, since these recover a high number of orthologous loci that resolve phylogenetic relationships.

## 1. Introduction

High-throughput DNA sequencing technologies (HTDNAs) significantly improve denser molecular sampling of DNA than Sanger-based capillarity technology. This increment of whole genomes published either as fully assembled and annotated genomes, or as raw data with poor or null informatics processing, has positively impacted advances in examining fundamental questions in flowering land plants. Fortunately, most of these massive DNA data are being deposited in free-access digital reservoirs, allowing the world’s scientific community to use them freely. In the particular case of angiosperms, most problems studied with HTDNAs are those of a phylogenetic nature. Presently, the term phylogenomics is used when a high number of loci (i.e., dozens and even hundreds) from DNA/RNA are sampled in a single or in various genomes (i.e., chloroplast, mitochondria, and nucleus) contained in plants. In land-flowering plants, phylogenomic analysis based on chloroplast genomes [1,2] and transcriptomes [3] has allowed scientists to resolve the relationships of early divergent lineages of angiosperms. The different genome-scale analyses converged in a topology of the species tree in which three orders—Amborellales (Amborellaceae, *Amborella trichopoda*), Nymphaeales (Cabombaceae, Hydatellaceae, and Nymphaeaceae), and Austrobaileyales (Austrobaileyaceae, Schisandraceae and Trimeneaceae)—were, successively, the sister lineages to the whole-monophyletic speciose group of angiosperms [1,2,3]. Recently, the old problem of determining the origin of angiosperms was reexamined in the broader phylogenetic study of Li et al. [4]. This study integrated previously published data obtained using HTDNAs, as well as other taxa de novo sequenced by the authors, in order to obtain 80 loci of the genome of the chloroplasts (>82,286 bp) of 2351 ingroup flowering land species. This study concluded that angiosperms emerged in the early Jurassic, which conflicts with the pollen fossil records data that indicate that angiosperms arose in the Cretaceous period. The results of this study revamped an old discussion with those fossil records defenders [5], and it is a fact that massive sequencing data will alter our views of the evolution of the biodiversity of our planet; they could even break scientific paradigms.

In addition, HTDNAs are also fertile tools for those scientific questions focused on smaller scales, either focused on the evolutionary divergence processes that occurred in particular flowering groups or in the biota evolution of local areas. Moreover, phylogenomic sampling may drastically increase the phylogenetic resolution yet at lower taxonomic hierarchical levels and in those plant groups that recently diverged [6,7,8]. The benefits of having a fully resolved phylogenetic tree species impact many areas: the inherent value is that phylogenetic relationships are clarified—often, in a phylogenetic tree, the diverging process may be inferred, which eventually may help to understand patterns of richness around the planet at different taxonomic hierarchical levels (i.e., orders, families, and genera). Furthermore, a fully resolved phylogeny may support a solution to those pendant taxonomic problems and the species boundaries can be clearly delimited.

Today, it is a fact that HTDNAs are a strategy that may provide denser molecular sampling, and for many research groups around the world, these technologies are a real available methodological alternative. Currently, to access this denser molecular sampling, there are different techniques that include: transcriptomic sequencing, low coverage sequencing (i.e., genome skimming), restriction-site-associated DNA sequencing (e.g., RAD-Seq), and target enrichment (studies cited in McKain et al. [9] and Yu et al. [10]). For the target-enrichment approach, one way is for the scientific team to start from zero in order to design the specific targeted probes (i.e., probe sets, probe kits, enrichment probes, and enrichment kits) for the studied plant taxa [7,11,12]; however, to begin, they will need to generate the primary data, which must be represented by at least a single whole-genome sequence or transcriptome from a taxon. If such primary data were already sequenced with free access, the scientific team would save money and time. If not, researchers would need to generate this primary set of DNA data and, consequently, would require more resources to experimentally produce the primary DNA/RNA data. In this scenario, the scientific team would have to design, essay, and fully complete experimental tests for a set of taxa; however, they would be dealing with complicated bioinformatics analyses. An alternative strategy is to use some of the available predesigned universal targeted probe sets and experimentally test the effectiveness of these on the studied taxa. These probe sets are presumed to be of universal efficiency and are expected to find dozens or hundreds of nuclear orthologous loci across different angiosperm taxa; however, these results are unknown until the experimental and bioinformatics processes are completed. Lastly, we propose here an alternative strategy that consists of testing in silico the available universal angiosperm probe sets, without preliminary experimental costs. This in silico test requires the availability of primary massive DNA sequences. It is expected that in this quantity of DNA, the nuclear genomes of the angiosperms of interest are represented, and thus it is expected that a high number of variable and orthologous loci will be recovered. In this in silico test, the research team must uniquely establish a detailed and appropriate bioinformatic protocol that can be implemented with free-access software, as in this study.

The objective of the present study was to compare the effectiveness in silico of two probe sets that promise universal results for angiosperms. The probe set Angiosperm v.1 was designed from the genomes of 25 species and contains 56,862 probes, expected to recover up to 517 individual exons with a maximum total length of 150 kbp [13]. In contrast, the probe set Angiosperms353 was designed from the transcriptomes of over 600 species and contains 75,151 probes, which are expected to recover 353 coding sequences (CDS) composed of one or more exons with a total length of 260 kbp [14]. These two universal probe sets have been applied in specific studies in various lineages of angiosperms for phylogenetic purposes. For example, the Angiosperm v.1 probe set was successfully used in the species of the genera *Aristolochia* [15] and *Acer* [16]. Recently, the probe set Angiosperms353 resolved phylogenetic issues in the genera *Nepenthes* [17], in the species of the genus *Cyperus* [18], and in the species of the ten families that comprise the order Cornales [19]. The results of these studies showed that these two probe sets were appropriate alternatives for obtaining loci that were used to construct resolved phylogenetic trees.

Accordingly, in this study, we tested in silico these two probe sets (Angiosperm v.1 and Angiosperms353) in ten Caryophyllids species: nine species of cacti (Cactaceae) and the species *Amaranthus hypochondriacus* (Amaranthaceae), used in the phylogenetic analysis as an external group. We focused on cacti species because phylogenetic studies carried out with these taxa repeatedly result in unresolved phylogenetic trees [20,21,22]. This lack of phylogenetic resolution may be due to the fact that most of these studies used poor molecular sampling (1–12 loci), or may be due to other factors, such as the recent postulation of the origin of Cactaceae (30 to 35 Ma) based on the molecular-clock model [23,24]. Moreover, it has been hypothesized that a global aridification that occurred nearly ∼12 million years ago (the late Miocene epoch) promoted the main evolutionary divergence in the Cactaceae family [23]. Consequently, the relatively recent origin of the Cactaceae family and the rapid and recent divergence of cacti species may cause a weak and shallow molecular separation, which has been reflected in those unresolved phylogenetic tree species. In addition, as HTDNAs have shown a significant increase in the number of loci-resolved phylogenetic tree species even in the taxa that have recently diverged [8], we are expecting that these two probe sets have a similar effectiveness in recovering nuclear orthologous loci that serve the cacti species analyzed in the phylogenetic studies.

## 2. Materials and Methods

### 2.1. Genomes of the Species Analyzed

At the beginning of our study, we carried out preliminary tests with the genomes <12 Gbp; however, the bioinformatic protocol from the pipeline used here did not work. For this, we e-searched all cacti species represented by >15 Gb of massive DNA sequencing in the NCBI digital database. A total of eight genomes of different cacti species were downloaded from the SRA repository. These DNA data ranged from 17 Gb (*Pereskia humboldtii*) to 57 Gb (*Selenicereus undatus*) (Table S1). In addition, the complete genome of the small globose cactus *Mammillaria huitzilopochtli* was de novo sequenced (raw data are available in S.S.) based on the PE genomic library sequenced in NovaSeq of Illumina. These nine cacti species are grouped into three cacti subfamilies and five tribes. The subfamily Cactoideae was represented by the tribes Cacteae (*M. huitzilopochtli*), Cereeae (*Cereus fernambucensis*), Hylocereeae (*Selenecereus undatus*), and Pachycereeae (*Carnegiea gigantea*, *Lophocereus schottii*, *Pachycereus pringlei*, and *Stenocereus thurberi*). The subfamily Opuntioideae was represented by the tribe Opuntieae (*Opuntia sulphurea*), and the subfamily Pereskioideae by the species *P. humboldtii*. In addition, *Amaranthus hypochondriacus* (Amaranthaceae, Caryophyllales; accession number in genebank, SRR2106212) was used as an external group for phylogenetic analyses of the studied species.

### 2.2. Bioinformatic Process to Test In Silico the Two Probe Sets

The massive amount of available raw data of each of the ten species (i.e., ten genomes) were firstly filtered by reads using Trim Galore version 0.6.7 [25] for subsequent bioinformatic analyses. For this, the total of the reads of each genome was trimmed, and the adapters were removed; the only reads used in the analysis were those that had a PHRED quality score ≥15 and a length ≥80 bp. The reads per genome that passed this filtering were in silico tested with the original predesigned targeted DNA sequences (TDS) from the two probe sets that were compared. Since the probe set Angiosperm v.1 includes 517 TDS, and Angiosperms353 includes 353 TDS, a total of 870 TDS were tested individually per genome following the pipeline available in HybPiper version 1.3.1 [26]. Accordingly, each of the 870 TDS were individually mapped and assembled with the main script (read_first.py) of this pipeline. The target files used in HybPiper correspond to the sequences from which the probe sets were designed and were downloaded from https://github.com/mossmatters/Angiosperms353/blob/master/Angiosperms353_targetSequences.fasta (Angiosperms353) (accessed on 15 March 2022) and https://www.biorxiv.org/content/10.1101/086298v2.supplementary-material (Angiosperm v.1) (accessed on 15 March 2022). Since the sequences of Angiosperm v.1 were 517 alignments in Phylip format, they were converted to fasta format, the gaps were removed, and the sequences were merged. Two scripts in HybPiper (scripts get_seq_lengths.py and hybpiper_stats.py) were used to obtain the length of the coding sequences (i.e., exons) and the statistical values that estimate the recovery efficiency of each of the two probe sets per genome; lastly, the R script (gene_recovery_heatmap.R) was used to plot the heatmaps and to visualize the efficiency of the recovering loci per genome. Finally, the script retrieve_sequences.py from the pipeline in HybPiper was used to generate the multifasta files to recover the total number of orthologous nuclear loci per genome. To avoid a possible bias in paralogous identification, we extracted the largest exon from each gene of the Angiospems353 dataset, and we generated the target file (Supplementary File S1) that contains the individual exons of 3829 of the 4781 CDS (target instances) contained in the Angiosperms353 dataset [14]. This target file was used to run an independent HybPiper pipeline, and the paralogous genes were added to the paralogs identified in the analysis of the complete CDS from Angiosperms353. All the sequences identified as paralogous loci in Angiosperm v.1 and Angiosperms353 (CDS + exon) were discarded for the phylogenetic analysis. The relative effectiveness of each of the two probe sets was estimated per species based on the following parameters: (1) the total number of TDS recovered (i.e., the number of loci) by each probe set; (2) the length (bp) of each locus was summed to have the total length (bp) of all the recovered loci; (3) the total number of paralogous loci was discarded for phylogenetic analysis; and (4) the proportion of parsimoniously informative sites (PIS) was obtained from the alignments of the DNA sequences of the orthologous loci.

### 2.3. Phylogenomics Analysis

The phylogenetic trees were based on the total nuclear orthologous loci recovered by Angiosperms353 (240 loci) and Angiosperm v.1 (71). The alignment of the species of each locus was carried out with MAFFT version v7.310 [27]. We observed a large disparity in the distribution of the orthologous loci among the ten studied species, and in order to diminish the missing data for phylogenetic analysis, we chose those loci that were recorded in the alignments of at least seven species. In addition, for each orthologous locus, its respective proportion of PIS was estimated with the program AMAS [28]. The application ModelFinder [29], implemented in IQ-TREE2 version 2.1.4-β [30], was used to select the partitioning scheme and to estimate the substitution models for each partition and for each locus. The DNA sequences of the orthologous loci obtained with each probe set were concatenated to construct the phylogenetic species-trees with IQ-TREE2 that ran 1000 replicates of an ultrafast bootstrap (UFBoot). In order to obtain the gene concordance factors (gCF) for each node of each of the two phylogenetic trees, we constructed an individual gene tree for each locus with IQ-TREE2.

## 3. Results

### 3.1. Effectiveness of the Two Probe Sets

**Figure 1:**
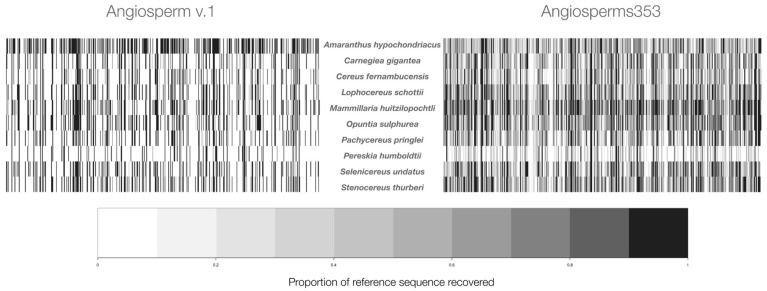
Heatmap per species is presented by raw data. To the left of the species names are the results obtained with Angiosperm v.1, and to the right, the results obtained with Angiosperms353. The gaps (white color) are caused by the absence of vertical lines, which indicates that the targeted DNA sequences did not find those loci in the genome tested.

The results showed that, among the ten studied species, the two probe sets tested had clear differences in the global efficiency of the TDS captured (Figure 1, Table 1). For the nine cacti species, Angiosperms353 showed a higher efficiency than Angiosperm v.1, which is supported by the total number of loci recovered (Figure 1), the length of the DNA sequences recovered by the locus, and the number of paralogous loci that were discarded (Table 1). These differences in global efficiency are visualized by the abundant dark lines seen in the heatmap for each species (Figure 1). Each vertical line indicates that an individual TDS was found in the tested genome, and its darkness indicates the proportion of the length of this sequence that was captured (Figure 1). For example, for *A. hypochondriacus*, the probe set Angiosperms353 showed a higher efficiency in capturing loci (316; Table 1); however, the length of the 216 loci captured with Angiosperm v.1 was larger (68,205 bp; Table 1); therefore, the heatmap is darker for this species (Figure 1).

The respective heatmaps of the cacti species show abundant gaps with Angiosperm v.1, because a large number of its 517 targeted DNA sequences did not match (Figure 1) in these genomes. This poorer capture of Angiosperm v.1 was documented in the four parameters used to compare its effectiveness to Angiosperms353. The results showed that, in the ten studied species, the probe set Angiosperms353 captured the highest number of nuclear orthologous loci (Table 1). In the ten studied species, with Angiosperms353, the average number of the orthologous loci was 275.6 ± 41.3 SD, and with Angiosperm v.1, it was 148.4 ± 49.6 SD. Moreover, in the nine cacti species studied, the length of the exons with Angiosperms353 was approximately four times larger than with Angiosperm v.1 (Table 1). In the ten studied species, the number of orthologous loci identified with Angiosperms353 was higher than with Angiosperm v.1 (Table 1). Additionally, in the ten studied species, Angiosperm v.1 recovered a total of 70 paralogous loci, and the percentage of these loci varied from 2.9% (*P. humboldtii*) to 20.3% (*M. huitzilopochtli*). In contrast, paralogous loci in Angiosperms353 varied from 0.4% (*C. fernambucensis*) to 6.7% (*M. huitzilopochtli*).

**Figure 2:**
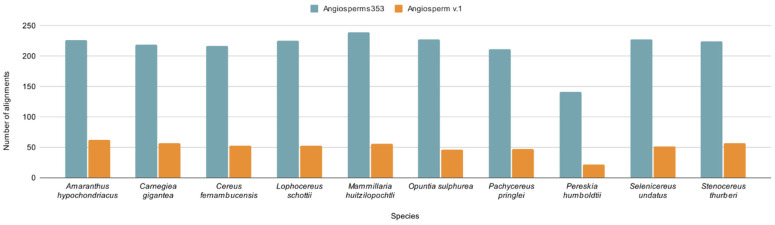
Total number of alignments obtained with Angiosperms353 and Angiosperm v.1 per species.

The orthologous loci identified in 70% of the analyzed species totaled 240 alignments with Angiosperms353, and only 71 alignments were obtained with Angiosperm v.1. The lowest number of alignments was obtained for the species *P. humboldtii*: 141 and 22 alignments obtained with Angiosperms353 and Angiosperm v.1, respectively. In contrast, the highest number of alignments was obtained for *A. hypochondriacus* (62) and *M. huitzilopochtli* (239) with Angiosperm v.1 and Angiosperms353, respectively (Figure 2).

With respect to the number of parsimoniously informative sites (PIS), Angiosperms353 recovered 4.32% of PIS in the sequences of the total number of orthologous loci aligned, whereas Angiosperm v.1 recovered 3.49% of PIS in such alignments.

### 3.2. Phylogenomics Analysis

**Figure 3:**
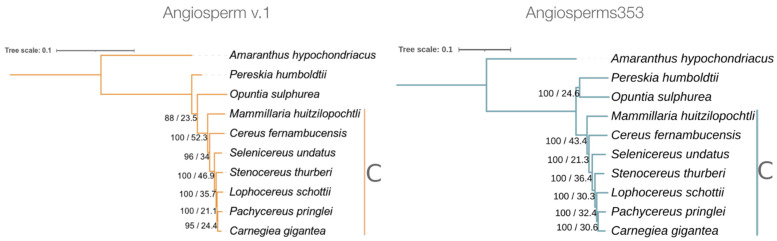
Phylogenetic trees obtained with ML for the nine studied cacti species; the prince’s feather (*A. hypochondriacus*) was the outgroup. The tree constructed with data from Angiosperm v.1 was based on 71 loci (20,082 bp), and that constructed with data obtained from Angiosperms353 was based on 262 loci (186,973 bp). The numbers to the side of the branches correspond to UFBoot (above slash) and gCF (below slash) values. The species grouped in the subfamily Cactoideae are indicated by the letter C.

The phylogenetic trees constructed with the DNA datasets provided by each of the two probe sets showed a fully resolved topology (Figure 3). Interestingly, in these two trees, the seven species of the subfamily Cactoideae were clearly grouped in a single monophyletic node. However, these trees showed subtle differences in the topology of the position occupied by *O. sulfurea*, as well as in the statistical values of the UFBoot and in the gCF. In the tree constructed from the data of Angiosperms353, the nodes were 100% supported by UFBoot values, but in the tree of Angiosperm v.1, these values varied from 88 to 100%. However, these two trees showed low values of gene concordance (<53%) for all the nodes.

## 4. Discussion

Our results showed that the two probe sets tested here were useful for Caryophyllids. However, Angiosperms353 had notably better results than Angiosperm v.1 for the nine cacti species, irrespective of the tribe or the subfamily in which these species are classified. It is evident that this dissimilar effectiveness was not related to the larger number of targeted DNA sequences (517) included in Angiosperms v.1 vs. 353 of Angiosperms353. We consider that these superior results obtained with Angiosperms353 for the cacti species could be explained by factors involved in the design process of the targeted DNA sequences for these probe sets: (1) the relatively wider taxonomic sampling employed to design Angiosperms353 (over 600 taxa) vs. Angiosperm v.1. (25 taxa), which could enhance the probability of sampling a larger number of targeted loci dispersed across different lineages, and that (2) Angiosperms353 was designed to recover up to 260 kbp of exons, whereas Angiosperm v.1 was designed to recover only 150 kbp.

Although these two probe sets were designed to recover only single copy genes, they failed because paralog sequences were recovered, but a substantially lower percentage was discarded with Angiosperms353. In addition, the species *P. humboldtii* showed the lowest global effectiveness with the two probe sets. We consider that these results may be caused by the relatively small quantity of the massive DNA dataset (17 Gb) available for this species, and it is probable that the nuclear genome was not completely sampled. Thus, it is important to consider the size of the nuclear genome in order to choose the appropriate platform for massive sequencing of the interested species.

With respect to *A. hypochondriacus*, the better results were obtained with Angiosperm v.1, except for the parameter of the number of paralogous loci. We consider that this was caused by the fact that, during the design of the targeted sequences of Angiosperm v.1, the authors sampled *β vulgaris* (Chenopodiaceae), a species that is phylogenetically close to the Amaranthaceae family, according to previous studies [31,32]. Consequently, we believe that the taxonomic identity of the taxa sampled during the design of the targeted sequences may impact the success of posterior studies of the predesigned probe sets.

Although, in this study, the primary objective was not to resolve the phylogenetic relationships of cacti species, the phylogenetic trees obtained allowed us to make some considerations. The two probe sets provided data used to obtain trees with similar topologies accompanied by low values of gCF. Consequently, these values of gCF indicated a high gene-tree discordance, and we believe that this was not caused by the number of loci used, since the number of loci obtained with the Angiosperms353 dataset (240) was higher than that obtained with Angiosperm v.1 (71). It is probable that these gCF values are related to the poor taxonomic sampling included in our study; however, previous phylogenomic studies with a broader taxonomic sampling also obtained high levels of gene-tree discordance [32,33]. Among the two trees, there was a punctual difference in the position occupied by *O*. *sulphurea* with respect to the species grouped in the Cactoideae subfamily, similar to the tree obtained with Angiosperm v.1 in previous phylogenetic studies [32,33,34]. However, based on the global parameters used to measure effectiveness, we consider that Angiosperms353 provided superior results for the cacti phylogenetic studies to Angiosperm v.1.

## 5. Conclusions

We concluded that the universal probe sets tested here are a confident strategy for studies that require a high number of single copy orthologous loci. We recommend that researchers compare previous in silico analyses of the selected probe sets in order to estimate their effectiveness. We should mention that, in this study, we focused on exons; thus, the molecular variation of introns was not explored. For introns, we would expect larger variation levels. It is likely that the molecular variation contained in introns may separate the more recent evolutionary processes that occurred at intraspecific levels. Lastly, our results confirmed that non-specific probe sets should not necessarily diminish the molecular sampling across different angiosperm groups. In fact, in a previous study carried out with the taxa of the genus *Cyperus* (Cyperaceae), the Angiosperms353 probe set provided a similar proportion of PIS compared to that obtained with a family-specific probe set, producing a similar resolution power at infrageneric taxonomic levels [18].

## Figures and Tables

**Table 1:** Comparison of the four parameters used to estimate the effectiveness between the two probe sets in silico tested. The results outside parentheses correspond to results obtained with Angiosperm v.1, and those inside parentheses with Angiosperms353. Total number of loci recovered with each probe set; number of loci that found a proportion ≥50% of the length of the targeted DNA sequence; the total length (bp) of sequences identified as exons and the quotient calculated; total length of exons from Angiosperms353/length of exons from Angiosperm v.1; and the absolute number of paralogous loci identified per species.

Species	Number of Loci Recovered	Number of Loci with >50% Sequence Length	Total Length of Exons (bp): Quotient	Number of Paralogous Loci
*A. hypochondriacus*	264 (316)	256 (167)	68,205 (65,658):0.96	24 (8)
*C. gigantea*	129 (270)	123 (165)	32,133 (124,773):3.88	12 (8)
*C. fernambucensis*	132 (269)	125 (145)	30,822 (116,277):3.77	13 (1)
*L. schottii*	137 (287)	135 (194)	35,127 (140,259):3.99	18 (6)
*M. huitzilopochtli*	177 (319)	168 (245)	43,875 (174,285):3.97	36 (21)
*O. sulphurea*	155 (292)	149 (204)	40,611 (150,720):3.71	18 (8)
*P. pringlei*	123 (253)	121 (158)	31,683 (122,844):3.88	6 (4)
*P. humboldtii*	68 (173)	67 (69)	18,885 (65,658):3.48	2 (2)
*S. undatus*	154 (287)	149 (170)	37,866 (133,071):3.51	16 (6)
*S. thurberi*	145 (290)	143 (204)	37,656 (143,322):3.81	16 (8)

## Data Availability

Not applicable.

## Supplementary Materials

The following are available online at https://www.mdpi.com/article/10.3390/genes13040570/s1, Table S1: List of the studied cacti species and the subfamily and tribes in which they are classified. Supplementary File S1: Target file with 3829 individual largest exons that were recovered from the total of the 4781 CDS (target instances) contained in Angiosperms353 dataset.
