# Supplementary material for: Effectiveness of Two Universal Angiosperm Probe Sets Tested In Silico for Caryophyllids Taxa with Emphasis on Cacti Species
Source: Genes (Basel). 2022 Mar 24;13(4):570. doi: 10.3390/genes13040570 (PMC9032312; doi:10.3390/genes13040570)
Supplement: Supplementary file 1 [file genes-13-00570-s001.zip › genes-1631672-supplementary.pdf]

Table S1. List of the studied cacti species and the subfamily and tribes in which they are classified. The accession number of the raw data of each genome was downloaded from the SRA available in NCBI (https://www.ncbi.nlm.nih.gov/sra). The amount of DNA sequenced data available in Giga base pairs (Gbp). NA indicates that this species is not assigned to some taxonomic tribe.

**Species name**	**Subfamily**	**Tribe**	**Accession number**	**Gbp**
1. *Carnegiea gigantea*	Cactoideae	Pachycereeae	SRR5036296	36
2. *Cereus fernambucensis*	Cactoideae	Cereeae	SRR10397793	44
3. *Lophocereus schottii*	Cactoideae	Pachycereeae	SRR5137211	37
4. *Mammillaria huitzilopochtli*	Cactoideae	Cacteae	(*de novo* sequenced)	50
5. *Opuntia sulphurea*	Opuntioideae	Opuntieae	SRR14865765	22
6. *Pachycereus pringlei*	Cactoideae	Pachycereeae	SRR5137214	30
7. *Pereskia humboldtii*	Pereskioideae	NA	SRR5137212	17
8. *Selenicereus undatus*	Cactoideae	Hylocereeae	SRR13710968	57
9. *Stenocereus thurberi*	Cactoideae	Pachycereeae	SRR5137213	37
